# Histomorphologic Profile of Skin Tumors

**DOI:** 10.31729/jnma.3988

**Published:** 2018-12-31

**Authors:** Rupendra Thapa, Pranita Gurung, Suspana Hirachand, Sanju Babu Shrestha

**Affiliations:** 1 Department of Pathology, Kathmandu Medical College Teaching Hospital, Sinamangal, Kathmandu, Nepal; 2 Department of Dermatology, Kathmandu Medical College Teaching Hospital, Sinamangal, Kathmandu, Nepal

**Keywords:** *benign*, *histopathological*, *malignant*, *tumors*

## Abstract

**Introduction:**

Skin tumors are not uncommon in the Nepalese community. Accurate diagnosis and categorization of these into different types is important because of its effect on prognosis and management. We conducted this study to find out the frequency of different histological types of skin tumors, correlate with their clinicopathologic profile and to determine the stage of malignant tumors.

**Methods:**

This is a descriptive cross-sectional study of skin tumors during the period February 2015 to January 2017. All the patients who were subjected to skin biopsy for skin tumors were included in this study. The biopsies taken were fixed in 10% formalin and then processed. Four microns thick sections were taken and stained with Haematoxylin and Eosin stain (H&E).

**Results:**

Total of 108 cases of skin tumors were included of which 68 (62.97%) cases were histologically diagnosed as benign and 40 (37.03%) cases were diagnosed as malignant tumors. Keratinocytic tumors were predominant among both benign and malignant skin tumors.

**Conclusions:**

Histopathological examination is the gold standard for accurate diagnosis and prognostic assessment of the patient. Staging of the malignant tumors in excision biopsy specimen needs to be emphasized since it is a predictor of prognosis.

## INTRODUCTION

Skin tumors comprises a wide variety of neoplasms of skin and subcutis that are further classified as keratinocytic, melanocytic, appendageal, hematolymphoid, and soft tissue tumors.^1^ The incidence of skin tumors varies widely in different part of the world. The incidence of cutaneous squamous cell carcinoma and other carcinomas of the skin varies globally, but is thought to be increasing overall since the 1960s at a rate of 3–8% per year.^2^ The high incidence of cutaneous squamous cell carcinoma and basal cell carcinoma is thought to be mostly the result of sun exposure and mutagenic effects of ultraviolet (UV) light.^3^

Benign tumors are sometimes confused clinically with malignancy, particularly when they are pigmented or ulcerated or inflamed, and histologic examination of biopsy specimen is required to establish a definite diagnosis. Hence biopsy of skin tumors is the gold standard for accurate diagnosis and prognostic assessment of the patient. The knowledge of histopathological patterns can help in prognosis and planning an effective management.^4^

This study was undertaken to determine the frequency of different histological types of tumors of the skin, correlate with their clinicopathologic profile and to determine the stage of malignant tumors so as to prevent mortality and morbidity associated with it.

## METHODS

This is a descriptive cross-sectional study of skin tumors carried out in the Department of Pathology, Kathmandu Medical College Teaching Hospital, Sinamangal, Kathmandu during a period of February 2015 to January 2017. All the patients who were subjected to skin biopsy for skin tumors were included in this study. The biopsies taken were fixed in 10% formalin and then processed. Four microns thick sections were taken and stained with Haematoxylin and Eosin stain (H&E). The tumors were classified according to World Health Organization classification of tumors-skin tumors 2006.^1^ Pathological staging of the malignant skin tumors was done according to the TNM system (American Joint Committee on Cancer 2010) only in excision specimen.^5^

Sample size required for the study was calculated by the following formula:


$$\begin{array}{l} \text{Sample size (n)}={\text{z}}^{2}*(\text{pq}) /{\text{e}}^{2}\\ =1.65*1.65*0.5*(1-0.5)0^{.12}\\ =68 \end{array}$$

where, z= 1.65 for CI=90%.

p= prevalence proportion in target population to have certain character

q= (1-p)

e= allowable error

The data collected wa entered in Microsoft Excel and analysed in SPSS (Statistical Packages for Social Services). The descriptive statistical analysis was done.

## RESULTS

A total of 108 skin tumors were analyzed during the study period. Spectrum of different histomorphological diagnosis of skin tumors was observed (Table 1).

**Table 1:** Distribution of cases according to histomorphological diagnosis.

S. N.	Histomorphological diagnosis	No.of cases n
	BENIGN TUMORS	68 (62.97)
	Keratinocytic tumors	30 (27.78)
1.	Seborrhoeic keratosis	17 (15.74)
2.	Verruca vulgaris	8 (7.40)
3.	Squamous papilloma	2 (1.86)
4.	Keratoacanthoma	3 (2.78)
	Melanocytic tumors	21 (19.44)
5.	Intradermal nevus	18 (16.67)
6.	Compound nevus	3 (2.78)
	Appendageal tumors	13 (12.03)
7.	Pilomatricoma	4 (3.70)
8.	Tricholemmoma	3 (2.78)
9.	Syringocystadenoma papilliferum	2 (1.86)
10.	Chondroid syringoma	1 (0.93)
11.	Cylindroma	1 (0.93)
12.	Poroma	1 (0.93)
13.	Sebaceoma	1 (0.93)
	Soft tissue tumors	4 (3.70)
14.	Angiokeratoma	2 (1.86)
15.	Dermatofibroma	1 (0.93)
16.	Capillary hemangioma	1 (0.93)
	MALIGNANT TUMORS	40 (37.03)
	Keratinocytic tumors	29 (26.86)
17.	Squamous cell carcinoma	17 (15.74)
18.	Basal cell carcinoma	12 (11.11)
	Melanocytic tumors	9 (8.33)
19.	Malignant melanoma	9 (8.33)
	Appendageal tumors	2 (1.86)
20.	Proliferating tricholemmal tumor	1 (0.93)
21.	Sebaceous carcinoma	1 (0.93)
	Total	108 (100)

Out of 108 skin tumors, 68 tumors were benign and 40 tumors were malignant. The benign tumors were more common than malignant tumors with benign to malignant ratio of 1.7:1. Among the benign tumors 41 cases were female and 27 cases were male with female to male ratio of 1.5:1. Female preponderance was observed in benign tumors but there was male preponderance in malignant tumors where 27 cases

were male and 13 cases were female with male to female ratio of 2.07:1 (Table 2).

**Table 2:** Age and gender distribution of Malignant tumors (n = 40).

S.N.	Malignant tumors	<40 years	41–50 years	51–60 years	61–70 years	71–80 years	Total n (%)	M	F
1.	Squamous cell carcinoma	0	1	4	9	3	17 (42.5)	12	5
2.	Basal cell carcinoma	1	2	3	5	1	12 (30)	7	5
3.	Malignant melanoma	0	0	3	5	1	9 (22.5)	7	2
4.	Sebaceous carcinoma	0	0	1	0	0	1 (2.5)	1	0
5.	Proliferating tricholemmal tumor	0	1	0	0	0	1 (2.5)	0	1
	Total	1	4	11	19	5	40 (100)	27	13

Age of the study population ranged from 16 to 80 years. The peak incidence was seen between 41 to 50 years in benign tumors where as in malignant tumors it was seen in between 61 to70 years (Table 2). Clinically most cases presented as nodules and ulcerated swelling. Head and neck region, particularly the face was commonly involved (Table 3).

**Table 3:** Site of involvement by different skin tumors.

S.N.	Skin tumors	No.of cases	Site of involvement
1	Seborrhoeic keratosis	17	Scalp (5), Neck (4), Back (3), Chest wall (3), Leg (2)
2	Verruca vulgaris	8	Face (3), Neck (2), Foot (2), Arm (1)
3	Squamous papilloma	2	Lip (1), Back (1)
4	Keratoacanthoma	3	Neck (2), Chin (1)
5	Intradermal nevus	18	Face (7), Neck (4), Arm (3), Back (4)
6	Compound nevus	3	Face (3)
7	Pilomatricoma	4	Neck (3), Back (1)
8	Tricholemmoma	3	Scalp (2), Neck (1)
9	Syringocystadenoma papilliferum	2	Neck (1), Back (1)
10	Chondroid syringoma	1	Face (1)
11	Cylindroma	1	Neck (1)
12	Poroma	1	Inguinal region (1)
13	Sebaceoma	1	Postauricular region (1)
14	Angiokeratoma	2	Back (1), Vulva (1)
15	Dermatofibroma	1	Chest wall (1)
16	Capillary hemangioma	1	Back (1)
17	Squamous cell carcinoma	17	Face (5), Lip (3), Scalp (2), Arm (2), Chest wall (2), Leg (3)
18	Basal cell carcinoma	12	Nose (4), Cheek (3), Forehead (3), Lip (2)
19	Malignant melanoma	9	Foot (4), Thigh (2), Arm (1), Toe (1), Scalp (1)
20	Proliferating tricholemmal tumor	1	Scalp (1)
21	Sebaceous carcinoma	1	Eye lid (1)

Amongst the benign category maximum number of cases comprised of keratinocytic tumors 30 cases (27.78%) and in malignant category also it was keratinocytic tumors 29 (26.86.5%) cases (Table 1). Out of 40 malignant skin tumors squamous cell carcinoma was the most common tumor comprising of 17 (42.5%) cases followed by basal cell carcinoma 12 (30%) cases, malignant melanoma 9 (22.50%) cases, proliferating tricholemmal tumor 1 (2.5%) case and sebaceous carcinoma 1 (2.5%) case (Table 2). As per TNM staging used in our study majority of squamous cell carcinomas were in pT2 stage whereas majority of basal cell carcinomas were in pT1 stage. In case of malignant melanoma majority were in stage pT4b (Table 4).

**Table 4:** Histological staging of malignant tumors as per TNM staging.

S.N.	Staging		No. of cases		Total
		Squamous cell carcinoma	Basal cell carcinoma	Malignant melanoma	
1	pT1	3	6	0	9
2	pT2	7	2	0	9
3	pT3b	0	0	1	1
4	pT4b	0	0	4	4
	Total	10	8	5	23

## DISCUSSION

Keratinocytic tumors are an important public health problem, despite their comparatively low mortality rate. There is an increasing incidence of squamous cell carcinoma of the skin in some countries. Keratinocytic tumors account for approximately 90% or more of all skin malignancies, of which approximately 70% are basal cell carcinomas.^1^ In view of various studies on skin tumors it seems that the incidence of squamous cell carcinoma is more in our part of the world.^6–9^ In present study the keratinocytic tumors, both benign accounting for 30 (27.78%) cases and malignant accounting for 29 (26.86%) cases were the common tumors of skin. Among the malignant keratinocytic tumors majority was squamous cell carcinoma 17 (15.74%) cases followed by basal cell carcinoma 12 (11. 11%) cases. The demographic characteristics of the patients in current study were relatively similar to a number of studies which describe a peak incidence of malignant skin tumors in the age 61–70 years and preponderance in male with male to female ratio of 2.07:1.^6,7,10^

In current study, the incidences of benign and malignant tumors were 68 (62.27%) cases and 40 (37.03%) cases. This findings were comparable the study done by Har-Shai et al. and Rajinder Kaur et al.^11,12^

In our study majority of squamous cell carcinomas were in pT2 stage whereas basal cell carcinomas were in pT1 stage. In case of malignant melanoma, majority were in stage pT4b as shown (Table 4). It has been said that BCCs rarely require staging given their minimal potential for metastasis. However, cutaneous SCC has a 4% annual incidence of metastasis, so staging is vital to its management and treatment.^13^

The location of SCC and BCC in our study was seen predominantly on face and nose which was in concordance with study done by Koyuncuer A. Ulceration was seen in majority of cases in current study which was also observed in the above mentioned study.^14^ Due to cosmetic purpose patient would like to remove lesions of the face so this could be one of the reason for the face being the common site of presentation in our study. Among the benign tumors intradermal nevus 18 (16.67%) cases was the predominant tumors which is comparable with the study done by Gundalli S et al.^10^

## CONCLUSIONS

This study revealed a wide spectrum of neoplastic lesions of skin. Majority of cases were benign tumors and among the malignant tumors, squamous cell carcinoma was predominant followed by basal cell carcinoma. Staging of the malignant tumors in excision biopsy specimen especially for squamous cell carcinoma needs to be emphasized since it is a predictor of prognosis.

## Conflict of Interest

**None.**
